# Nudging to move: a scoping review of the use of choice architecture interventions to promote physical activity in the general population

**DOI:** 10.1186/s12966-019-0844-z

**Published:** 2019-09-03

**Authors:** S. Forberger, L. Reisch, T. Kampfmann, H. Zeeb

**Affiliations:** 1 Leibniz Institute for Prevention Research and Epidemiology – BIPS, Achterstraße 30, 28359 Bremen, Germany; 2 Leibniz-Chair, Leibniz Institute for Prevention Research and Epidemiology – BIPS, Achterstraße 30, 28359 Bremen, Germany; 3 Copenhagen Business School, MSC, Dalgas Have 15, 2000 Frederiksberg, Denmark; 4 Health Sciences Bremen, University of Bremen, Bremen, Germany

**Keywords:** Physical activity, General population, Nudge, Choice architecture, Behavioural insights, Behaviourally informed policy

## Abstract

**Background:**

Nudges are used to alter lifestyles and thus curb the rise of non-communicable diseases. Physical activity is a core prevention strategy to reduce the burden of non-communicable diseases. This paper aims to (1) give an overview of the scope of interventions using choice architecture techniques to promote physical activity at the population levels and (2) identify research gaps by analysing the different approaches in terms of class and type of intervention used.

**Methods:**

A systematic electronic database search was combined with snowball citation sampling of a starter set of publications to search for studies published through October 2018 reporting interventions to promote physical activity at the population level using choice architecture techniques. The methodology of the Joanna Briggs Institute for Scoping Reviews was applied.

**Results:**

In all, 35 publications were included. Most of the interventions used point-of-choice prompts tested at railway stations, shopping malls and airports (*N* = 27). Eight studies were online studies. While all studies were aimed at the general population, details, if reported at all, were vague and basic. All studies focused on individual-level lifestyle behaviour. None of the studies attempted to alter population-based lifestyle behaviour. Online and “real-world” approaches were rarely combined. Neither, interventions targeting meso- and macro-level structures nor combinations of individual-level and specific meso- or macro-level interventions were found.

**Conclusion:**

Nudging is in principle an effective approach to promote physical activity within the general population. However, there are large gaps in research. Available opportunities have not yet been exhausted. Further research is needed that is explicitly based on behavioural insights and covering the full range of nudging approaches, particularly focussing on theoretical developments, practical feasibility tests and scale-up activities.

**Electronic supplementary material:**

The online version of this article (10.1186/s12966-019-0844-z) contains supplementary material, which is available to authorized users.

## Background

Since the publication of the seminal book *Nudge* by Thaler and Sunstein in 2008 [1], the use of behavioural insights in general and nudges in particular has gained increasing interest among public and private institutions. The Organisation for Economic Co-operation and Development (OECD) concludes in its 2017 report that the use of behavioural insights goes beyond a trend towards widespread dissemination [2] as it is studied in various areas and integrated into policy making [3, 4]. The Organisation for Economic Co-operation and Development defines behavioural insights as a mix of “traditional economic strategies with insights from psychology, cognitive sciences and other social sciences to discover the many irrational factors that influence decision making” [5]. Governments are increasingly interested in using behaviourally informed policies to shape citizens’ behaviour as a complement to or replacement of traditional instruments such as bans or mandates [2–4, 6]. Behavioural insights help to understand why people often fail to act in their best interest, to follow well-informed preferences or to achieve their set goals. Nudges can help to overcome these challenges by using the same habits, biases or boundaries to alter our decision-making in favour of the more preferred behaviour [7]. According to Thaler and Sunstein, nudges aim to “alter people’s behaviour in a predictable way without forbidding any option or significantly change their economic incentives. To count as a mere nudge, [an] intervention must be easy and cheap to avoid. Nudges are not mandates” [1] and “allow [s] them [people] to go their own way” [8]. Nudges alter the underlying “choice architecture”, the context in which the decision is made. Their appeal lies in the idea of influencing individual decision-making with minimal effort in order to support or achieve behavioural changes [9]. The concept has attracted the attention of governments at all levels interested in influencing lifestyles at the population level by focusing directly or indirectly on the physical and social environment [10, 11], especially with the constraint of limited public resources. This is attractively combined with the preservation of individual freedom of choice at a time when individualisation is prominent and the one-fits-all approach increasingly rejected [12]. The promising aspect of choice architecture approaches is the idea that once the right stimulus is found, individuals will automatically choose a “better” (e.g. healthier, more sustainable, more environmentally friendly, more financially attractive) alternative [13, 14] rendering expensive enforcement structures obsolete.

It is increasingly apparent that systematic changes in people’s environments (micro-, meso-, and macro-level) where people make decisions can be important catalysts for changing behaviour at individual and population-level [15–17]. Micro-, meso- and macro-level refer to a general approach to analysis with micro-level (individual-level) covering the individual in his or her social setting or a small group of individuals in a particular context. Examples include but are not limited to: persons, citizens, families and households. Meso-level (social-organisational level) analyses fall between the micro- and macro-levels, normally covering the community or an organisation. It could also be a whole village, town or federal state. The macro-level, sometimes referred to as institutional level, includes among others the analytical units: states, nations, or societies [18, 19]. However, systematic research analysing the use of choice architecture approaches to effect change on the micro-, meso- and macro-levels is only slowly emerging, especially for meso- and macro-level interventions. Since their introduction the terms choice architecture and nudge have been used in different ways, across a wide range of interventions, multiple behaviours, environmental contexts (e.g. physical activity [20–22], and food consumption [23–25] or levels of analysis [15, 26–30]). In most cases, intervention approaches were grouped based on common mechanisms like behavioural economics, gamification elements, or point-of-choice [24, 26, 31–36]. To date, there have been few systematic overviews on the effectiveness of selected choice architecture interventions in specific areas such as food consumption [23, 25, 36, 37], pro-environmental behaviour [38], or lifestyle risk factors [32]. Szaszi et al. gave a domain-general overview of the used choice architecture intervention in the domains consumer choice, health, sustainability, education, transport, finance, health, and other [39]. To categorize the intervention used, Szaszi and his colleagues used the classification suggested by Münscher et al. [40].

Münscher and his colleagues distinguish between three categories of choice architecture techniques: (A) decision information covering various techniques that target the presentation and provision of decision-relevant information without changing existing options. The second category (B) decision structure refers to techniques aimed at designing options and associated consequences such as pre-selected options for organ donations or pension schemes (default option) or 5-cent taxes for shopping bags. The last category (C) decision assistance includes techniques to support and implement intentions to change a given behaviour by encouraging engagement, feedback, or reminders (Table 1).

**Table 1:** Choice architecture categories and techniques according to Münscher et al. (2016) with examples

Category	Technique	Examples
A Decision information	A1 Translate information Includes: reframe, simplify	Reframing call for blood donations as death-preventing rather than life-saving
	A2 Make information visible Includes: make own behaviour visible (feedback), make external information visible	Feedback about one’s own behaviour (fitness tracker), information in the form of graphics, etc. about e.g. house insulation, credit card statements, calorie intake
	A3 Provide social reference point Includes: refer to descriptive norm, refer to opinion leader	Information about the behaviour of people from one’s own peer group or people who are valued for special purposes, experts, or role models
B Decision structure	B1 Change choice defaults Includes: set no-action default, use prompted choice	Pre-selected options that leave the freedom to select a different option (or not) such as done for organ donation or pension savings in some countries (default options), poster/banners to use stairs
	B2 Change option-related effort Includes: increase/decrease physical/financial effort	(Re) arrangements of food items in grocery stores, of menu cards in restaurants, or the presentation of food dishes at buffets so that the healthier choices are easier to reach/to choose
	B3 Change range or composition of options Includes: change categories, change grouping of options	Segregating healthy options into diverse categories
	B4 Change option consequences Includes: connect decision to benefit/cost, change social consequences of the decision	5-cent tax for a shopping bag, possibility to take part in a lottery when complying with medication or taking part in a survey
C Decision assistance	C1 Provide reminders	Get reminders
	C 2 Facilitate commitment Includes: support self-commitment/public commitment	www.stickk.com, browser apps, blocking the internet access for specific items; agreements between parents and schools

Hollands et al. present another approach to classify choice architecture interventions aiming to alter micro environments in order to induce certain behaviours [15, 41]. They differentiate six intervention types: availability, position, functionality, presentation, size, and information with an intervention focus on product related objects or the wider environment. While the first version covered food, alcohol, tobacco, and physical activity, the final version of TIPPME does not cover physical activity (Table 2). Sunstein gave an overview of the ten main nudges currently used [8] (Table 3).

**Table 2:** TIPPME intervention typology for environments to change behaviour according to Hollands et al. (2017)

Class	Intervention type	Examples for intervention focus		
		Product	Related objects	Wider environment
Placement	Availability	Adding non-alcoholic options to a bar’s range of drinks, or removing less healthy snack options from a vending machine	Add baskets, trolleys or trays to a shop or restaurant to increase the number of products that people can select and carry	Removing some of the entrance doors leading to a bar or cafeteria
	Position	Place less healthy options further away from seating, entrance, or main thoroughfare	Move refrigerators containing sugary drinks to a less convenient location in a supermarket	Move dividing walls or fixed furniture to alter layout of a supermarket, restaurant, or bar
Properties	Functionality	Allowing easier opening or pouring or demarcate plate to provide guidance for amounts of vegetables vs. meat selected	Demarcate shopping trolley space to indicate designated space for fruit and vegetables	Alter functionality of entrance and exit doors (e.g. change their opening mechanism)
	Presentation	Plain packaging for cigarettes or alcohol products	Colours, textures, and visual design of shelf displays, menus, and other related object	Indoor climate: temperature, humidity, air pressure, lighting
	Size	Change size of portions, plates, packages	Change size of shopping trolleys or baskets, cafeteria trays, or food and drink storage equipment	Size and shape of windows, or fixed furniture
	Information	Health warnings on cigarette packets, alcohol consumption units on glasses	Nutritional information on menus or menu boards	Information on posters, leaflets, or computer screens, in the wider environment

**Table 3:** The 10 most important Nudges (Sunstein, 2014)

Nudge		Example
1	Default rules	Automatic enrollment in programs, including education, health, savings
2	Simplification	In part to promote participation in existing programs
3	Uses of social norms	“Most people pay their taxes on time.”, “Nine out of ten hotel guests reuse their towels.”
4	Increases in ease and convenience	Making low-cost options or healthy foods visible
5	Disclosure	Economic or environmental costs associated with energy use, or the full cost of certain credit cards
6	Warnings, graphic or otherwise	Pictures on cigarette packages
7	Pre-commitment strategies	Pre-commit to engaging in certain activities such as smoking cessation
8	Reminders	Email or text message, as for overdue bills and coming obligations or appointments
9	Eliciting implementation intentions	“Do you plan to vaccinate your child?”
10	Informing people of the nature and consequences of their own past choices	Expenditures on health care or on electric bills

This scoping review aims to give an overview of work in a specific part of this growing field of research: the application of choice architecture interventions to increase physical activity in the general population, i.e. not in a specific setting or target group but in the population as a whole. The specific feature of this scoping review is to provide an overview of research in the field of physical activity promotion for the general population, to map the literature, and to identify knowledge gaps. Objectives of systematic reviews such as the feasibility, appropriateness, rationality, or effectiveness of interventions will not be addressed. In our scoping review, we have included studies explicitly stating that they use choice architecture interventions. In addition, we searched grey literature including reports of the Organisation for Economic Co-operation and Development (OECD), the World Health Organisation (WHO), the European Union (EU), and the United Nations (UN) to focus on population-level physical activity interventions. Although physical activity has been shown to be effective in preventing many chronic diseases [42, 43] physical activity in the population remains low [44]. Population-based approaches which explicitly include context and environmental factors in the decision-making process could represent a valid option to increase physical activity in daily life. However, little is known so far about choice architecture interventions to target physical activity in the general population.

To date, in the field of physical activity most work has been done for specific target groups [45, 46], specific settings [46, 47], disease prevention [48–50], and technology use [51–53]. There have been activities focusing on public policy evolution to promote physical activity [54–56] and some studies were conducted with a special focus on financial incentives without a focus on nudging [57–62]. In 2010, Nocon et al. and Soler et al. published papers analysing the effectiveness of point-of-choice prompts [22, 63]. Zimmerman et al. [21] examined behavioural economics with the aim to promote physical activity. He suggested looking beyond the default option, i.e. pre-selected options, and to focus instead on “anchors” that are reference points (e.g. norms, framing, habits) influencing subsequent judgements in order to see how they interact with the context and influence preferences [21]. However, little is known beyond the application of point-of-choice nudges in terms of population-level interventions for the promotion of physical activity. This scoping review aims to address this knowledge gap by [1] providing an overview of the scope of choice architecture interventions to promote physical activity within the general population and [2] analysing the different approaches in terms of class and type of intervention used.

## Methods

The scoping review is based on the Joanna Briggs Institute methodology for Scoping Reviews [64] using the preferred reporting items for systematic reviews [65] and the transparent reporting of systematic reviews and meta-analyses “PRISMA” flowchart. Although scoping reviews follow a similar approach to systematic reviews [66], they differ in terms of objectives and key characteristics. They answer broader questions that go beyond those related to the effectiveness of treatments or interventions and aim to (a) map existing literature in terms of nature, features and volume, (b) clarify work definitions and conceptual boundaries and (c) identify gaps in the existing literature and research [67].

### Literature search

Medline, PsycInfo, different Web of Science databases (Science Citation Index, Arts & Humanities Citation Index, Conference Proceedings Citation Index, Social Science & Humanities Index, Book Citation Index–Science, Book Citation Index–Social Science & Humanities), CINHAL, Econ. Lit and ASSIA were used to search for studies published through October 2018 reporting interventions to promote physical activity in the population using choice architecture techniques. Databases and publication period covered for each database are shown in Table 4.

**Table 4:** List of databases and their characteristics

Database		Provider	Time span
1	Medline	PubMed	1982–10/2018
2	PsycInfo	Ovid	1806–10/2018
3	Science Citation Index Expanded	Web of Science	1900–10/2018
	Social Science Citation Index		1956–10/2018
	Arts & Humanities Citation Index		1975–10/2018
	Conference Proceedings Citation Index–Science		2013–10/2018
	Conference Proceedings Citation Index–Social Science & Humanities		2013–10/2018
	Book Citation Index–Science		2013–10/2018
	Book Citation Index–Social Science & Humanities		2013–10/2018
4	CINHAL Econ.Lit	EBSCO	1937–10/2018 1886–10/2018
5	ASSIA	Proquest	1987–10/2018

Inclusion was restricted to full-text papers and to studies published in English or German

### Search strategy

The search strategy consisted of a combination of keywords, Medical Subject Headings (MeSH-Terms) and Thesaurus of Psychological Index Terms. We combined five search themes with Boolean operators (Table 5). An example of the search is available in Additional file 1. The search strategy was developed and tested in collabrotation with a research librarian beforehand.

**Table 5:** List of search themes and terms used for the search strategy

Search themes		Search terms	Search type
1	Behavioural insights	Behavioural insight, nudging, nudge, behavioural economic, behavioural public policy, choice architecture, choice intervention, behavioural informed	Title/Abstract
2	Physical activity	Sport, sporting, exercise, physical fitness, physical activity, aerobic, training	Title/Abstract
3	Physical activity	Exercise, sports, physical education and training	MeSH-Terms
4	Walkability	Walkability	MeSH-Terms
5	Active transport	Active transport	MeSH-Terms

Consideration of: British and American English, singular/plural

The reference lists of the included studies were searched to identify additional studies. Hand-searched grey literature was included if eligible (see “Screening procedure” below). The starter-set publications for the grey literature research were the reports of the Organisation for Economic Co-operation and Development (OECD), the United Nations, the European Commission, and the World Bank about using behavioural-informed strategies [2, 4, 68–70], as well as the World Health Organization Health-enhancing physical activity (HEPA) policy audit tool (PAT) (WHO HEPA PAT) country reports. Following a snowball approach, we first used backward snowballing, using the reference lists to identify new papers. The identified papers were examined based on inclusion and exclusion criteria for the systematic literature search. Second, forward snowball sampling was applied to include studies based on papers citing the paper under examination [71]. The process was repeated until no more new papers were found. For the forward snowball sampling system, Google Scholar was used.

### Screening procedure and inclusion/exclusion criteria

Following the literature search, the screening procedure was based on predefined inclusion and exclusion criteria (Table 6) and consisted of two consecutive phases. All publications were independently screened by two reviewers. First, titles and abstracts were screened to exclude articles that did not meet the eligibility criteria. In a second step, full texts were independently reviewed and evaluated by two reviewers. Any disagreements were either resolved by consensus or by discussion with a third reviewer. In terms of publications, we included those that applied choice architecture techniques. We included full-text papers in English and German. We included peer-reviewed publications as well as grey literature to incorporate results that may not yet have been published. If the full text was not available we contacted the authors. We excluded review articles but screened the reference lists of the papers to identify suitable publications. Study designs included in the review were: randomised controlled trials (RCTs), non-randomised controlled trials as well as observational studies aimed at the general population. Studies that did not aim for the general population, e.g. those targeted at specific groups such as students or staff or settings such as schools, workplaces, day care centres, or nursing homes, were not considered. We included online studies, i.e., studies that recruited their participants through open online platforms, provided they excluded only persons under 18 years of age and had no further restrictions.

**Table 6:** Inclusion and exclusion criteria

Inclusion criteria	
1	Full-text paper.
2	The language of the paper was English or German.
3	The paper was published in a peer-reviewed journal or as grey literature.
4	The studies in the paper investigated one or more behaviourally informed intervention techniques attributed to a nudge or connected to the choice-architecture literature.
5	The target group was the general population.
Exclusion Criteria	
1	Review articles, conference abstracts and conference papers. However, we screened the review articles for suitable publications.
2	Intervention targeting specific settings (schools, kindergartens, workplaces), specific target groups (children, workplace staff members, women), or disease prevention/weight-management programs.

### Data extraction

Only full texts were analysed for data extraction. Retrieved publications were organised via a reference management software and later managed with Rayyan [72]. All included publications were analysed and data were extracted into an a priori developed extraction form (Table 7), which was tested beforehand on three studies to ensure all relevant results were extracted [73, 74]. Data extraction was conducted independently by two authors. In case of disagreement a third author was consulted.

**Table 7:** Predefined data extraction form with categories of the extracted data and characteristics of the categories

Category		Characteristics
1	Author	All authors of the publication
2	Year	Year of publication
3	Publication type	Journal article, report, grey literature
4	Domain/subdomain	While the domain of physical activity (PA) is growing the category summarises the aim of the intervention indicating specific subdomains if needed, such as active transport, cycling, walking and stair use.
5	Origin	The country in which the intervention was carried out
6	Aim of the intervention	Aim of the intervention
7	Study design	Study design used
8	Intervention	Short description of the intervention
9	Setting	Setting of the intervention
10	Target group specifications	Details reported about the target group (inclusion/exclusion of persons, children, disabled persons)
11	Approach	Population-wide, individual-based
12	Analytical level	Micro, meso, macro
	Choice-architecture category and intervention techniques	Based on the taxonomy of Münscher, Vetter & Scheuerle (2015) the interventions were sorted into three choice-architecture categories with nine techniques: (A) Decision information (1) translate information (e.g. framing techniques and simplification of information), (2) make information visible (e.g. using feedback techniques and presenting external information), (3) provide social reference point (e.g. referring to a descriptive norm or to an opinion leader) (B) Decision structure (1) change choice defaults (e.g. opt-in, opt-out techniques or prompted choice), (2) change option-related effort (e.g. financial or physical effort), (3) change range or composition of options (e.g. changing categories or grouping of options), (4) change option consequences (e.g. changing social consequences of the decision or connecting it to benefit or cost) (C) Decision assistance (1) provide reminders (e.g. making information more salient or easier to access), and (2) facilitate commitment (e.g. supporting self or public commitment to choice).
14	Typology of interventions with special focus on micro environment	Typology of intervention in accordance with TIPPME [26] Placement Availability Position Properties Functionality Presentation Size Information
15	Intervention results	Study results

The extracted data were synthesised and presented in a table mapping the publications included against the predefined categories (Table 7). To categorise the interventions two taxonomies were combined. The choice architecture techniques used were categorised by the taxonomy developed by Münscher et al. [40] and sorted into three choice-architecture categories with nine techniques. The categories include: (A) decision information ((A1) translate information (A2) make information visible, (A3) provide social reference), (B) decision structure ((B1) change choice defaults, (B2) change option-related effort, (B3) change range or composition of options, (B4) change option consequences), (C) decision assistance, ((C1) provide reminders, (C2) facilitate commitment) (Table 1). In addition, interventions explicitly used to alter micro environments, that means the context in which the intervention is applied, were categorised in accordance with TIPPME [15, 41] which focuses on the categorisation of interventions aiming to change micro environments in order to change behaviour. The interventions were classified by placement and properties with six interventions types (availability, position, functionality, presentation, size, information) (Table 2).

## Results

### Search results

**Fig. 1:**
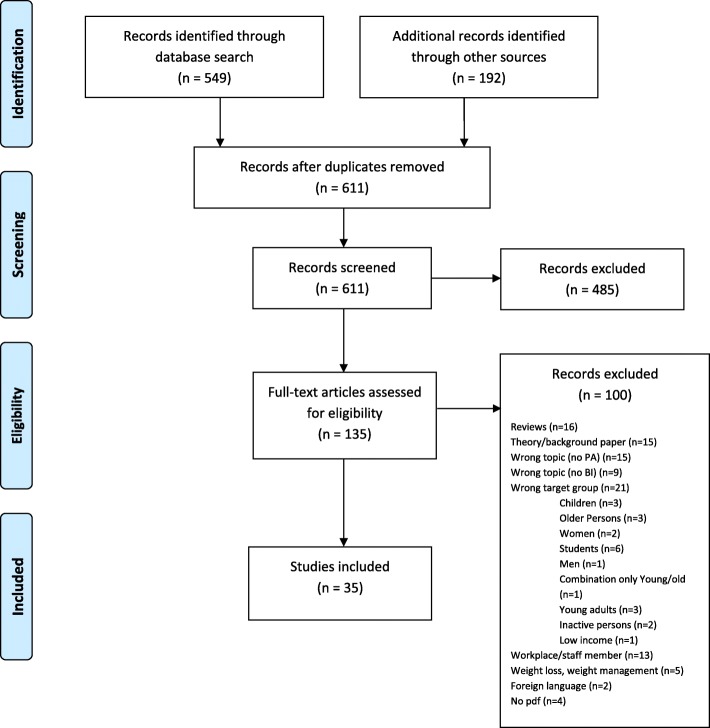
Study Flow Diagram

In the database search phase, 549 articles were retrieved. A total of 192 records were found by an additional search beginning with the starter-set publications. After controlling for duplicates, 611 records were included in the screening of abstracts and titles. During that screening, 485 articles were excluded because they did not meet the inclusion criteria. In the next step, 135 full-text articles were assessed for eligibility. At the end of screening, 35 articles met the selection criteria for the review (Fig. 1, Additional file 2).

### Characteristics of the selected articles

All studies described in the selected articles focus on the increase of physical activity with the following specifications: increase of stair use [75–100], exercise commitment [101–103], physical activity in general [104–107], and walkability (step count) [108]. All articles focused on the general population. However, in the studies that reported data on the general population, children and persons with children, disabled persons, persons with luggage and, in online studies, persons under the age of 18 were excluded. Most studies were conducted in the UK [77, 81, 84–89, 92, 95, 105], followed by online studies mostly without geographical focus [101–104, 106–108], and studies from the USA [76, 78, 79, 97, 98, 109], and Hong Kong [75, 80]. There was one study each from Singapore [96], Japan [91], Denmark [83], Sweden [82], Germany [90], Belgium [100], France [99], South Africa [93], and Spain [94]. Most studies were conducted in train stations/underground train stations [78, 79, 81–83, 89–92, 94, 96, 99, 100], followed by studies in malls/shopping malls [76–78, 84–88, 95, 100], airports [79, 97, 98, 109], pedestrian transit systems [80, 82], two public buildings [79, 93], and one bus station [78]. Eight studies were conducted online only [101–108]. Most of the studies used an observational study design [76–83, 85–90, 92, 95–97, 100, 109]. Experimental study [99, 101, 104, 107], post-test study [102], interrupted time-series, randomised controlled trial [75, 103, 105], interview study [84], quasi-experimental [94, 98, 108], and cross-sectional survey [91] designs were represented as well. All interventions targeted the micro level. None of the studies reported any involvement of communal, regional, or national authorities (Table 8).

**Table 8:** Characteristics of included articles (*n* = 35)

Characteristic	Number of articles	Percent
Geographical region		
UK	10	31
Online	8	20
USA	7	17
Hong Kong	2	6
Japan	1	3
Denmark	1	3
Sweden	1	3
Germany	1	3
Belgium	1	3
South Africa	1	3
Spain	1	3
France	1	3
Singapore	1	3
Setting (multiple settings possible)		
Train station/underground train station	12	34
Mall/shopping mall	10	29
Airport	4	11
Pedestrian transit system	2	6
Library (public building)	2	6
Bus station	1	3
Online only	8	23
Study design		
Observational study	21	60
Experimental study	4	11
2 × 2 Post-test study	1	3
Interrupted time-series design	1	3
Randomised controlled trial	2	6
Interviews	1	3
Quasi-experimental	3	9
Cross-sectional survey	1	3
Not specified	1	3
Intervention aim		
Increased stair use	27	77
Increase in exercise commitment	3	9
PA in general	4	11
Walking (step count)	1	3
Intervention level (micro, meso, macro)		
Micro	35	100
Choice-architecture category (Taxonomy following Münscher et al. 2016; multiple categories possible)		
A. Decision information category		
A1 Translation information (e.g. framing techniques)	3	9
A2 Making information visible	3	9
A3 Provide social reference points	2	6
B. Decision structure		
B1 Change choice defaults	30	86
B2 Change option-related effort	0	0
B3 Change range or composition of option	0	0
B4 Change option consequences	6	17
C. Decision assistance		
C1 Provide reminders	1	3
C2 Facilitate commitment	3	9

### Use of nudges for physical activity promotion in the general population

Most of the interventions used were nudges from the choice-architecture category B1: “Change choice defaults”. Within this category, 27 studies used point-of-choice prompts like placing banners or posters at stairs or escalators with different messages to encourage stair use [75–94, 96–100, 109, 110]. Three studies used default options [89–91]. Three studies used A1: “Translate information” with gain/loss message framing [102–104]. Three studies used the choice-architecture category A2: “Making information visible”, like using bio-feedback with the help of an external monitoring device [108], computer-generated tailored feedback of participants’ physical activity levels [107], or feedback on daily individual goal performance [105]. Two studies used A3: “Provided social reference points”, such as whether or not the personal physical activity level is in accordance with recommendations [107] or whether individuals using financial stakes are more successful [103]. Six studies used B4 “Change option consequences” by connecting options to small benefits or costs [101, 103–106, 108]. One study used reminders (C1) [107]. Three studies used C2: “Facilitate commitment” by giving the option to use challenges between participants, and allowing participants to track success or failure to comply or designate a referee [101, 105, 106].

While the studies using point-of-choice prompts mostly used one nudge, the eight online studies combined several choice-architecture categories. Four studies used two choice categories by:

- combining gain/loss message framing (A1) with a default option (B1) [102]
- combining framing (A1) and a “change option consequences” (B4; getting a greater reward when spending more time being physically active) [104];
- giving feedback with an external monitoring device (A2) and offering an opportunity to earn cents when reaching personalized daily step goals (B4) [108]; and
- combining reward (B4) and commitment structures (C2) by allowing challenge [106].

Two studies utilized three combinations:

- “change choice default” (B1), “change option consequences” (B4) and “facilitate commitment” (C2) [101]; and
- “make information visible” (feedback) (A2), “change option consequences” (B4) and “facilitate commitment” (C2) [105].

Two studies combined four nudges [103, 107].

In order to examine interventions altering micro environments in more detail we used the TIPPME typology that classifies interventions into “properties” and “placement” (Table 9). There were 22 studies that used only the intervention class properties and intervention type information by placing posters or banners to prompt stair use. One study used the intervention type placement and analysed the role of an opportunity for increased stair use by modifying the environment in favour of either escalator use (two escalators ascending) or stair use (only one escalator ascending) [82]. Six studies combined the two intervention classes. One intervention used a combination of two intervention types, information and size, using big letters for the information material [80]. Two studies alternated between posters and stair banners (information and position) [92]. Three studies used information and presentation, explicitly mentioning the use of colourful designs or yellow background [75, 80, 88, 95].

**Table 9:** Analysing prompted choice following TIPPME (Hollands et al., 2017)/ n = 27 (*n* = 8 online-only studies, taxonomy not applicable)

Intervention class	Intervention type	Number of studies
Properties	Information	22
Placement	Availability	1
Properties & placement	Information & position	2
	Information & presentation	3
	Information & size	1

*n* = 27 studies. However, Eves et al. [78] reported three sub-studies using point-of-decision prompts, one properties and information, two information and presentation in one paper, which were counted here separately

Almost all studies using point-of-choice prompts reported positive effects and an increase in stair use under intervention conditions; except for four studies [75, 80, 90, 92]. Eves and Masters found no effect in one of their studies in Hong Kong [80]. Three other studies [75, 90, 92] found mixed results. Nine studies reported post-intervention data: five studies reported no effect under the post-intervention condition [75, 78, 83, 96, 99] and four studies reported higher stair use after intervention compared to baseline [85, 90, 93, 94]. Six of the eight online studies reported positive effects of the interventions [101, 103, 105–108]. One study found no effect [102] and one had mixed effects [104]. Two online studies reported post-intervention data: Patel et al. reported a decrease in staircase use, but higher rates compared to baseline [105], and van’t Riet et al. reported no post-intervention effect [107].

## Discussion

The aim of this scoping review was to provide an overview of interventions for physical activity promotion that [1] use choice architecture approaches within the general population and [2] to classify the approaches used. Although we were able to identify several studies using this approach, the number of studies is surprisingly small compared to the attention given to nudging or physical activity. Further, there are no studies targeting meso- or macro-level structures to promote physical activity. This may be due in part to the complexity of the issue of physical activity promotion at the population level, encompassing governmental structures on various levels to target obesogenic environmental aspects [111].

The interventions reported used mostly point-of-choice prompts targeting the individual and encouraging pedestrians to use stairs. However, population characteristics were rarely reported in detail, which is also due to the focus of the studies. The studies used observations or unobtrusive technology targeting the general population in order to examine behaviour in real life that is important to analyse real-world effects. Exclusions were made on the basis of observations, which are subject to mistakes. When reported, children and persons with children, impaired persons, and persons with large bags were excluded, and the physical-activity behaviour of participants was not monitored. In light of habit formation and the change of a population norm or reframing physical activity within a culture as a whole, these excluded populations are of interest, as well [21]. It is known that physical activity is not only performed by individuals simply for the sake of health but more importantly to manage everyday life such as commuting to work, grocery shopping, visiting doctors, bringing up children, visiting friends or leisure time activities [112]. In a review, Westerterp reported that habitual physical activity in early life and during growth had a significant independent influence on the growth of a lean body mass, which by implication has a long-term effect on body mass and calorie expenditure at a later age [113]. Following this line, all population groups, also persons with children (role-model function) and children themselves should be considered when designing nudges for physical activity promotion. Further, in terms of participation and health equality, disabled persons have to be considered as well; otherwise the interventions used are not sufficient. Designs have to be used that allow the observation of all population groups and the combination of different types of intervention that work for the whole population.

The online studies were able to apply more than one nudge and combined, for example, “change choice defaults” with “change in option consequences”. However, we did not find any studies that utilised a combination of interventions targeting stair use at an individual level with posters/banners and supporting this with online tools, or using meso- or macro-level support for long-term interventions. Nor could we find work with a combination of targeting environmental cues for physical activity with nudges at the meso- or macro-level and technology support. There is no information in the studies about the support and cooperation of, for example, a mall, an airport, or a city management that allowed stair-use interventions. Also, there is no reference to governmental programs or actions that used behaviourally informed approaches. It could be argued that this gap exists because there are currently no such interventions combining these approaches or because they are not being researched. However, in order to more broadly influence the environmental context to foster daily physical activity as a cultural or socially accepted norm, structures at the organisational, community, federal, and/or state level have to be targeted to effect a change of attitudes and norms in favour of habitual physical activity [112, 114, 115]. This is also important because the studies have shown that point-of-choice prompts are mostly effective and therefore a cost-effective way to promote physical activity in everyday life as long as the interventions continue. However, few studies report data on stair use after the end of the intervention. Most studies reported that stair use decreased after the prompt was removed, and most of the stair-use interventions had no long-term effects [22, 63]. More research is needed to answer questions such as: What is the timeframe for a stair use intervention until habituation/tolerance will occur? Can the habituation effect be delayed? How can the effect be delayed? How can different intervention approaches be meaningfully combined (poster, banner, music, digital, technical installation for stair use games)? How can results based on this research be scaled-up? How can meso- and macro-level structures be addressed and which combinations of micro- (individual-level interventions) meso- and macro-level approaches affect the behaviour of the population and alter habitual physical activity? These questions open up a much broader field for further research using choice architecture techniques to promote physical activity for the general population [13].

With their work Münscher et al. [40] and Hollands et al. [15, 41] further developed initial taxonomies categorising choice-architecture approaches. However, while Münscher et al. is very useful to categorise choice architectures, TIPPME allows much more precision in defining the micro environments. A combination of both taxonomies may be advantageous to classify physical activity promotion. While the development of an own classification system for physical activity taking into account the combination of individual characteristics and role of environment should be considered, the use of two existing classifications allows for comparison between the research fields as soon as sufficient data is available.

The currently small number of studies makes it impossible to compare the results with other areas like food, alcohol use, or smoking [15, 32, 39] or to determine whether certain countries prefer and support specific behaviourally informed approaches [3, 116]. States and social systems may differ when it comes to the acceptance of behaviour-informed interventions and to the extent of their intrusiveness in everyday life. Initial work has been done to analyse the level of approval of nudges between groups of nations and the circumstances in which they are supported [117–120]. However, more work is needed to test which nudging approaches are most effective in combination with which state structures and related (social) norms for physical activity promotion, as the use of these approaches is always contextual and culture specific.

Our search and analysis aimed to be broad and inclusive in order to obtain an overview of approaches to physical activity promotion using choice architecture interventions and to concepts surrounding this effort. To our knowledge, no comparable work has been done so far. For the interpretation of the results, some points should be considered. First of all, interventions or broader policies that use nudging implicitly without stating so were not included. Only studies related to behavioural insights, choice architecture, nudging, behavioural economics and behaviourally informed intervention were included. Second, specific approaches relevant to physical activity promotion such as active transport and urban planning may have been overlooked with our search strategy and were not included in the scoping review if they did not explicitly refer to nudging or choice architecture. Furthermore, research reporting e.g. on the use of public transport or the role of urban planning for traffic management purposes or environmental reasons not related to physical activity was not included, although increased use of public transport could increase daily physical activity. Here, more sensitive research strategies that take into account the complexity of the issue, indirect effects and inter-dependencies of the different research fields through e.g. nested research strategies might be more appropriate. Finally, Embase was not included in the research because we did not have access. The scoping review was not pre-registered in PROSPERO, which covers only systematic reviews and a research protocol was not published beforehand. In order to ensure that reporting on the process and the results was as objective as possible the authors previously agreed on a publication proposal that included an abstract, the search strategy, and predefined inclusion and exclusion criteria. Furthermore, all screenings were carried out by two reviewers and the results were discussed throughout the group.

## Conclusion

Given the complexity of health intervention and the interplay among micro-, meso- and macro-level factors, the full potential of choice architecture interventions has yet to be realized. The results of this scoping review show that studies use nudging for physical activity promotion, but mostly on an individual level. There are large gaps in the approaches and instruments for increasing daily physical activity in the population. Studies are concentrating mainly on point-of-decision nudges. A few online studies combine several nudges but are limited to the online domain. A combination of online and “real-world” approaches has not yet been implemented. Furthermore, we found no interventions targeting meso- and macro-level structures, and a combination and linkage of individual-level intervention and specific meso- or macro-level intervention to nudge at the population level has not been applied so far. There is a lack of studies testing the use of behaviourally informed public policy making to enable sustainable changes in meso- and macro-level environmental factors. It is rarely attempted to scale-up the intervention from the individual level to the meso- or macro-level. Further tests of such approaches and the exploration of the full potential of choice architecture interventions for physical activity and more active mobility are needed.

## Additional files

Additional file 1: Characteristics of studies included. (DOCX 43 kb)

Additional file 2: PubMed search strategy. (DOCX 16 kb)

## Data Availability

All data generated or analysed during this study are included in this published article [and its supplementary information files].

## Abbreviations

HEPA PAT: Health-enhancing physical activity (HEPA) policy audit tool (PAT)
OECD: Organisation for Economic Co-operation and Development
PA: Physical activity
UN: United Nations
WHO: World Health Organization
