# Supplementary material for: Nudging to move: a scoping review of the use of choice architecture interventions to promote physical activity in the general population
Source: Int J Behav Nutr Phys Act. 2019 Sep 3;16:77. doi: 10.1186/s12966-019-0844-z (PMC6724306; doi:10.1186/s12966-019-0844-z)
Supplement: Supplementary file 1 — Characteristics of studies included. (DOCX 43 kb) [file 12966_2019_844_MOESM1_ESM.docx]

|  | **Author** | **Year** | **Origin/Country** | **Domain/ Subdomain** | **Setting** | **Target group specification/coding** | | | **Level of analyses** | **Choice-architecture category** | | | | | | | |  | **Intervention instruments used to alter properties or placement of objectives within micro-environments** |
| --- | --- | --- | --- | --- | --- | --- | --- | --- | --- | --- | --- | --- | --- | --- | --- | --- | --- | --- | --- |
|  |  |  |  |  |  |  |  |  |  | **(A) Decision information** | | | **(B) Decision structure** | | | | **C) Decision assistance** | |  |
|  |  |  |  |  |  | **Age** | **further coding** | **exclusion** |  | **(1) translate information** | **(2) make information visible** | **(3) provide social reference point** | **(1) change choice defaults** | **(2) change option-related effort** | **(3) change range or composition of options** | **(4) change option consequences** | **(1) provide reminders** | **(2) facilitate commitment** |  |
| **1** | Allais et al. | 2017 | France, Paris | PA/stair use | Metro | no information | no information | no information | micro |  |  |  | x (prompted choice) |  |  |  |  |  | Information (properties) |
| **2** | Andersen et al. | 1998 | USA, Baltimore | PA/stair use | Mall | >40 and <40 | gender, weight class (not overweight, overweight), ethnicity (black, white) | persons carrying a baby, child and those judged to be younger than 18 years of age | micro |  |  |  | x (prompted choice) |  |  |  |  |  | Information (properties) |
| **3** | Bellettiere et al. | 2018 | USA, San Diego Airport | PA/stair use | Airport | most common age category: 31–50 (49.3%), followed by 51–65 (23.9%), 18–30 (21.1%), and 66 or older (5.7%) | gender, ethnicity (black/white), overweight, college graduates | older than 18 | micro |  |  |  | x (prompted choice) |  |  |  |  |  | Information (properties) |
| **4** | Bhattacharya et al. | 2015 | online | PA/Increasing exercise by commitment contracts | StickK.com website | 18-75, average age 33 years | gender |  | micro |  |  |  | x (default) |  |  | x |  | x | Not applicable (online) |
| **5** | Blamey et al. | 1995 | UK, Scotland | PA/stair use | City centre underground station | no information | gender | carrying luggage, pushchairs | micro |  |  |  | x (prompted choice) |  |  |  |  |  | Information (properties) |
| **6** | Boen et al. | 2010 | Belgium, Hasselt | PA/stair use | Mall and 2 train stations | no information | no information | no information | micro |  |  |  | x (prompted choice) |  |  |  |  |  | Information (properties) |
| **7** | Brownell et al. | 1980 | USA | PA/stair use | Shopping mall, train station, bus station | over 30, under 30 years | gender, race (black/  white), weight (obese/non-obese) | persons carrying items larger than an attaché case, children | micro |  |  |  | x (prompted choice) |  |  |  |  |  | Information (properties) |
| **8** | Coleman & Gonzalez | 2001 | USA, El Paso | PA/stair use | Airport, bank, office building, University of Texas at El Paso [UTEP] library | no information | gender | no information | micro |  |  |  | x (prompted choice) |  |  |  |  |  | Information (properties) |
| **9** | de Bruijn et al. | 2014 | online | PA/exercise | n/a | medium age = 32.38 years | gender | no information | micro | x |  |  | x (default) |  |  |  |  |  | Not applicable |
| **10** | Eves & Masters | 2006 | Hong Kong | PA/stair use | Mid-Levels escalator system (pedestrian transit system) | 8.6% old, 2.1% children, 48.4% female, 18.4% non-Asian, 88.6% adults | gender, appearance of over 60 years old, ethnic grouping, walking up the travellator | no information | micro |  |  |  | x (prompted choice) |  |  |  |  |  | Information (properties) |
| **11** | Eves et al. | 2008 | Hong Kong | PA/stair use | Mid-Levels escalator system (pedestrian transit system) | no information | no information | no information | micro |  |  |  | x (prompted choice) |  |  |  |  |  | Information (properties)  Presentation (properties) in sub-study 2 and 3 |
| **12** | Evers et al. | 2008 | UK, Birmingham | PA/stair use | Train station with three platforms | no information | no information | no information | micro |  |  |  | x (prompted choice) |  |  |  |  |  | Information (properties)  Size (properties) |
| **13** | Faskunger et al. | 2003 | Sweden, Stockholm | PA/stair use | Train station | no information | no information | no information | micro |  |  |  | x (prompted choice) |  |  |  |  |  | Placement (availability) |
| **14** | Fulton et al. | 2017 | USA, Atlanta | PA/stair use | Airport | no information (data collection by sensors to count travellers) | no information | no information | micro |  |  |  | x (prompted choice) |  |  |  |  |  | Information (properties) |
| **15** | Goldhaber-Fiebert et al. | 2010 | online | PA/nudge to sustain increased exercise | Users of a free, web-based tool to create exercise contracts | 18-69, average age = 34 years,  female = 56%, non-US = 22% | gender, nationality | no information | micro | x |  |  | x (default) |  |  | x |  |  | Not applicable |
| **16** | Hsu & Vlaev | 2014 | online, US only | PA general | Recruited from Amazon Mechanical Turk | 18-70, median age 31 years | gender, weight (overweight) | US members only | micro | x |  |  |  |  |  | x |  |  | Not applicable |
| **17** | Iversen et al. | 2007 | Denmark, Copenhagen | PA/stair use | 2 train stations, Copenhagen | male/female = about 50-50% | gender | blind people, people carrying children or large luggage, rollators, canes or bikes | micro |  |  |  | x (prompted choice) |  |  |  |  |  | Information (properties) |
| **18** | Kerr et al. | 2000a | UK, Birmingham | PA/increase stair use | Shopping mall | no information | no information | no information | micro |  |  |  | x (prompted choice) |  |  |  |  |  | Information (properties) |
| **19** | Kerr et al. | 2001b | UK, Birmingham | PA/increase in stair use | Shopping centre & train station | no information | gender, age (grey hair and appear over 60 years old), children, luggage | no information | micro |  |  |  | x (prompted choice) |  |  |  |  |  | Information (properties)  Size (properties) |
| **20** | Kerr et al. | 2001c | UK | PA/increase in stair use | Shopping centre | no information | gender, age, ethnicity, children and presence of bags | no information | micro | x |  |  | x (prompted choice) |  |  |  |  |  | Information (properties)  Position (placement) |
| **21** | Kerr et al. | 2001d | UK, Midland region | PA/increased stair use | Shopping mall | 58% women, 42% men, 24% classified older than 60 (grey hair), 12% non-Caucasian | gender, children, adults, age (with grey hair and/or appearance over 60 years), ethnicity (non-Caucasians), presence of bags | no information | micro |  |  |  | x (prompted choice) |  |  |  |  |  | Information (properties) |
| **22** | Kerr et al. | 2001e | UK | PA/increase in stair use | 2 shopping malls | no information | gender, age, ethnicity | no information | micro |  |  |  | x (prompted choice) |  |  |  |  |  | Information (properties) |
| **23** | Lewis & Evers | 2012 | UK | PA/increase in stair climbing | Train station | no information | gender | physical incapacity, large bags (larger than a briefcase or medium-sized bag), accompanying children (head below shoulder height of accompanying adult) | micro | x |  |  | x (prompted choice) |  |  |  |  |  | Information (properties) |
| **24** | Mitchell et al. | 2018 | online | PA/increase in walking (step count) | Carrot Rewards App | mean age = 33.7 years | age, gender, province | 13+ | micro |  | x |  |  |  |  | x |  |  | Not applicable |
| **25** | Müller-Riemenschneider et al. | 2010 | Deutschland, Berlin | PA/increase in stair use | Underground train station | no information | gender | no information | micro |  |  |  | x (prompted choice) |  |  |  |  |  | Information (properties) |
| **26** | Nomura et al. | 2009 | Japan, Himeji City, Hyogo Prefecture | PA/increase in stair use | Commuter train station | over 65 under 65, students | gender, age (gray hair and/or an appearance of over 65 years old and gait), and junior high school and high school students (wearing student uniforms and/or an appearance of 13–18 years old) | children, Infants | micro |  |  |  | x (prompted choice) |  |  |  |  |  | Information (properties) |
| **27** | Olander et al. | 2008 | UK | PA/increase in stair use | Train station | no information | gender | travellers accompanied  by children (head below shoulder height of accompanying adult), persons carrying  large bags | micro |  |  |  | x (prompted choice) |  |  |  |  |  | Information (properties)  Position (placement) |
| **28** | Patel et al. | 2017 | USA, Framingham | PA | Online | mean age = 55.4 years | 18+ | children | micro |  | x |  |  |  |  | x |  | x | not applicable |
| **29** | Pillay et al. | 2009 | South Africa, Cape Town | PA/increase in stair use | Sports Science Institute of South Africa (SSISA) | students, staff, visitors | no information | no information | micro |  |  |  | x (prompted choice) |  |  |  |  |  | Information (properties) |
| **30** | Puig-Ribera & Eves | 2009 | Spain, Barcelona | PA/promote stair use | Underground stations | over and under 60 years | gender, white and non-white, people carrying bags larger than medium size | children | micro |  |  |  | x (prompted choice) |  |  |  |  |  | Information (properties) |
| **31** | Russell | 2002 | USA, regional Midwest airport | PA/increase in stair use | Airport | over and under 40 years | gender | physically challenged people, traveling with young children, more than 1 piece of luggage | micro |  |  |  | x (prompted choice) |  |  |  |  |  | Information (properties) |
| **32** | Sloan et al. | 2013 | Singapore, Singapore | PA/increase in stair use | Singapore Mass Rapid Transit (MRT) station | no information | gender | no information | micro |  |  |  | x (prompted choice) |  |  |  |  |  | Information (properties) |
| **33** | van Mierlo et al. | 2016 | online | PA | GOODcoins, a self-guided, free-to-consumer engagement and rewards platform | age (n) 19 and under = 70 20-29 = 438 30-39 = 322 40-49 = 254 50-59 = 106 60 and over = 30 unknown = 78 | gender, age, nationality | no information | micro |  |  |  |  |  |  | x |  | x | Not applicable |
| **34** | van ’t Riet et al. | 2010 (publication in advance 2009) | online province of Limburg in The Netherlands | PA, in general being active, following the Dutch recommendations | Online, www.health-alert.nl | 18 - 87 years, mean age = 46.3 years | gender, age, ethnicity, education | under 18 | micro | x | x | x |  |  |  |  | x |  | Not applicable |
| **35** | Webb & Eves | 2007 | UK | PA/increase in stair use | Shopping mall | 93.4% under 60 years | classified under 60 years or 60+ | no information | micro |  |  |  | x (prompted choice) |  |  |  |  |  | Information & Presentation (properties) |

1. Kerr, J., Eves, F., & Carroll, D. (2000). Posters can prompt less active people to use the stairs. *Journal of Epidemiology and Community Health, 54*(12), 942-943.
2. Kerr, J., Eves, F. F., & Carroll, D. (2001). The influence of poster prompts on stair use: The effects of setting, poster size and content. *British Journal of Health Psychology, 6*(4), 397-405.
3. Kerr, J., Eves, F. F., & Carroll, D. (2001). Getting more people on the stairs: The impact of a new message format. *Journal of Health Psychology, 6*(5), 495-500.
4. Kerr, J., Eves, F., & Carroll, D. (2001). Six-month observational study of prompted stair climbing. *Preventive Medicine, 33*(5), 422-427.
5. Kerr, J., Eves, F., & Carroll, D. (2001). Encouraging stair use: Stair-riser banners are better than posters. *American Journal of Public Health, 91*(8), 1192-1193.
