# Supplementary material for: Nudging to move: a scoping review of the use of choice architecture interventions to promote physical activity in the general population
Source: Int J Behav Nutr Phys Act. 2019 Sep 3;16:77. doi: 10.1186/s12966-019-0844-z (PMC6724306; doi:10.1186/s12966-019-0844-z)
Supplement: Supplementary file 2 — PubMed search strategy. (DOCX 16 kb) [file 12966_2019_844_MOESM2_ESM.docx]

**Additional file 2: PubMed search strategy**

This template search strategy was adapted as needed to fit the other databases searched for the scoping review. The exact search for each of the databases will be available on request from the authors at final publication.

General:

Database: PubMed

Article Types: All

Text availability: All (Abstract, full text, free full text)

Publication dates: All

Species: Humans

**Block A: Behavioural Insights**

| Search term | Search type | Results |
| --- | --- | --- |
| “behavioral insight*" OR "behavioural insight*" OR "nudging" OR "nudge" OR "behavioral economic" OR "behavioural economic" OR “behavioral economics” OR “behavioural economics” OR "behavioural public policy" OR "behavioral public policy" OR "behavioural public policies" OR "behavioral public policies" OR "choice architecture*" OR "choice intervention" OR "choice interventions" OR "behavioural informed" OR "behavioral informed” OR “economic incentive” OR “economic incentives” OR “economic instrument” OR “economic instruments” | Title/Abstract | 2700 |

**Block B: Physical Activity**

| Search term | Search type | Results |
| --- | --- | --- |
| sport OR sports OR sporting OR exercis* OR "physical fitness" OR "physical activity" OR "physical activities" OR aerobic* OR training* | Title/Abstract | 743648 |
| “Exercise” OR "Sports” OR "Physical Education and Training" | MeSH Terms | 257534 |

A AND B = 213

Search code:

(((((((“Exercise”[MeSH Terms] OR "Sports”[MeSH Terms] OR "Physical Education and Training"[MeSH Terms])))) OR (((sport[Title/Abstract] OR sports[Title/Abstract] OR sporting[Title/Abstract] OR exercis*[Title/Abstract] OR "physical fitness"[Title/Abstract] OR "physical activity"[Title/Abstract] OR "physical activities"[Title/Abstract] OR aerobic*[Title/Abstract] OR training*[Title/Abstract]))))) AND (((("behavioral insight*"[Title/Abstract] OR "behavioural insight*"[Title/Abstract] OR "nudging"[Title/Abstract] OR "nudge"[Title/Abstract] OR "behavioral economic"[Title/Abstract] OR "behavioural economic"[Title/Abstract] OR "behavioral economics"[Title/Abstract] OR "behavioural economics"[Title/Abstract] OR "behavioural public policy"[Title/Abstract] OR "behavioral public policy"[Title/Abstract] OR "behavioural public policies"[Title/Abstract] OR "behavioral public policies"[Title/Abstract] OR "choice architecture*"[Title/Abstract] OR "choice intervention"[Title/Abstract] OR "choice interventions"[Title/Abstract] OR "behavioural informed"[Title/Abstract] OR "behavioral informed”[Title/Abstract] OR "economic incentive"[Title/Abstract] OR "economic incentives"[Title/Abstract] OR “economic instrument” [Title/Abstract] OR “economic instruments"[Title/Abstract)))) Sort by: Best Match

**Block C: Walking**

| Search term | Search type | Results |
| --- | --- | --- |
| “walking” OR “active transport” | MeSH Terms | 48566 |

A AND C = 8

Search code:

(((((((((("behavioral insight*"[Title/Abstract] OR "behavioural insight*"[Title/Abstract] OR "nudging"[Title/Abstract] OR "nudge"[Title/Abstract] OR "behavioral economic"[Title/Abstract] OR "behavioural economic"[Title/Abstract] OR "behavioral economics"[Title/Abstract] OR "behavioural economics"[Title/Abstract] OR "behavioural public policy"[Title/Abstract] OR "behavioral public policy"[Title/Abstract] OR "behavioural public policies"[Title/Abstract] OR "behavioral public policies"[Title/Abstract] OR "choice architecture*"[Title/Abstract] OR "choice intervention"[Title/Abstract] OR "choice interventions"[Title/Abstract] OR "behavioural informed"[Title/Abstract] OR "behavioral informed”[Title/Abstract] OR "economic incentive"[Title/Abstract] OR "economic incentives"[Title/Abstract] OR "economic instrument" [Title/Abstract] OR "economic instruments" [Title/Abstract]))))) AND ( "0001/01/01"[PDat] : "2018/10/15"[PDat] ))) AND (("walking" OR "active transport") AND ( "0001/01/01"[PDat] : "2018/10/15"[PDat] ))) AND ( "0001/01/01"[PDat] : "2018/10/15"[PDat] ))) AND (("walking"[MeSH Terms] OR "active transport"[MeSH Terms]) AND ( "0001/01/01"[PDat] : "2018/10/15"[PDat] )) Sort by: Best Match Filters: Publication date to 2018/10/30
